# Deciphering RNA modification and post-transcriptional regulation with NetRNApan

**DOI:** 10.1093/bib/bbaf690

**Published:** 2025-12-26

**Authors:** Haodong Xu, Wankun Deng, Ruifeng Hu, Binfeng Liu, Wenchao Zhang, Lujuan Wang, Lin Qi, Xiaolei Ren, Chao Tu, Zhihong Li, Zhongming Zhao

**Affiliations:** Department of Orthopaedics, The Second Xiangya Hospital, Central South University, No. 139, Renmin Road, Changsha, Hunan 410011, China; Hunan Key Laboratory of Tumor Models and Individualized Medicine, The Second Xiangya Hospital of Central South University, No. 139, Renmin Road, Changsha 410011, China; Center for Precision Health, McWilliams School of Biomedical Informatics, The University of Texas Health Science Center at Houston, 7000 Fannin Street, Houston, TX 77030, United States; Center for Precision Health, McWilliams School of Biomedical Informatics, The University of Texas Health Science Center at Houston, 7000 Fannin Street, Houston, TX 77030, United States; Center for Precision Health, McWilliams School of Biomedical Informatics, The University of Texas Health Science Center at Houston, 7000 Fannin Street, Houston, TX 77030, United States; Department of Orthopaedics, The Second Xiangya Hospital, Central South University, No. 139, Renmin Road, Changsha, Hunan 410011, China; Hunan Key Laboratory of Tumor Models and Individualized Medicine, The Second Xiangya Hospital of Central South University, No. 139, Renmin Road, Changsha 410011, China; Department of Orthopaedics, The Second Xiangya Hospital, Central South University, No. 139, Renmin Road, Changsha, Hunan 410011, China; Hunan Key Laboratory of Tumor Models and Individualized Medicine, The Second Xiangya Hospital of Central South University, No. 139, Renmin Road, Changsha 410011, China; Department of Orthopaedics, The Second Xiangya Hospital, Central South University, No. 139, Renmin Road, Changsha, Hunan 410011, China; Hunan Key Laboratory of Tumor Models and Individualized Medicine, The Second Xiangya Hospital of Central South University, No. 139, Renmin Road, Changsha 410011, China; Department of Orthopaedics, The Second Xiangya Hospital, Central South University, No. 139, Renmin Road, Changsha, Hunan 410011, China; Hunan Key Laboratory of Tumor Models and Individualized Medicine, The Second Xiangya Hospital of Central South University, No. 139, Renmin Road, Changsha 410011, China; Department of Orthopaedics, The Second Xiangya Hospital, Central South University, No. 139, Renmin Road, Changsha, Hunan 410011, China; Hunan Key Laboratory of Tumor Models and Individualized Medicine, The Second Xiangya Hospital of Central South University, No. 139, Renmin Road, Changsha 410011, China; Department of Orthopaedics, The Second Xiangya Hospital, Central South University, No. 139, Renmin Road, Changsha, Hunan 410011, China; Hunan Key Laboratory of Tumor Models and Individualized Medicine, The Second Xiangya Hospital of Central South University, No. 139, Renmin Road, Changsha 410011, China; Department of Orthopaedics, The Second Xiangya Hospital, Central South University, No. 139, Renmin Road, Changsha, Hunan 410011, China; Hunan Key Laboratory of Tumor Models and Individualized Medicine, The Second Xiangya Hospital of Central South University, No. 139, Renmin Road, Changsha 410011, China; Center for Precision Health, McWilliams School of Biomedical Informatics, The University of Texas Health Science Center at Houston, 7000 Fannin Street, Houston, TX 77030, United States; MD Anderson Cancer Center UTHealth Graduate School of Biomedical Sciences, 6767 Bertner Avenue, Houston, TX 77030, United States; Human Genetics Center, School of Public Health, The University of Texas Health Science Center at Houston, 7000 Fannin Street, Houston, TX 77030, United States

**Keywords:** RNA modification, epitranscriptomics, m^5^U, m^6^A, deep learning, RNA binding protein, motif discovery

## Abstract

RNA modification, which is evolutionarily conserved, is crucial for modulating various biological functions and disease pathogenesis. High resolution transcriptome-wide mapping of RNA modifications has facilitated both data resources and computational prediction of RNA modification. While these prediction algorithms are promising, they are limited in interpretability or generalizability, or the capacity for discovering novel post-transcriptional regulations. Here, we present NetRNApan, a deep learning framework for RNA modification site prediction, motif discovery and trans-regulatory factor identification. Using m^5^U profiles generated by FICC-seq and miCLIP-seq technologies and single-base resolution m^6^A sites from multiple experiments as cases, we demonstrated the accuracy of NetRNApan with more efficient and interpretive feature representations. For m^5^U modification, we uncovered five representative clusters with consensus motifs that may be essential by decoding the informative characteristics detected by NetRNApan. Furthermore, NetRNApan revealed interesting trans-regulatory factors and provided a protein-binding perspective for investigating the function of RNA modifications. Specifically, we discovered 21 potential functional RNA-binding proteins (RBPs) whose binding sites were significantly linked to the extracted top-scoring motifs for m^5^U modification. Two examples are ANKHD1 and RBM4 with potential regulatory function of m^5^U modifications. Meanwhile, the analysis of convolution layer parameters within the model offers valuable insights into the regulation of m^6^A in humans. Collectively, NetRNApan demonstrated high accuracy, interpretability and generalizability for study of RNA modification and mRNA regulation. NetRNApan is freely available at https://github.com/bsml320/NetRNApan.

## Introduction

Since the discovery of the first RNA modification in 1957, ~170 distinct RNA modification types in various types of RNA molecules have been reported [1–5]. Recent advances in high-throughput RNA-sequencing methods have uncovered many important functions of RNA modifications, which play in a variety of biological processes. Consequently, there arises a new discipline, ‘epitranscriptomics’ [6–9]. For example, a set of RNA modifications, e.g. N^6^-methyladenosine (m^6^A) [10], 5-methyluridine (m^5^U) [11], 5-methylcytosine (m^5^C) [12], and N4-acetylcytidine (ac^4^C) [13], were characterized to affect RNA stability, translation, localization, and degradation [4]. The cellular transcriptome and proteome are shaped by all of these molecular perturbation events, affecting a series of cellular and developmental functions and linking to many human diseases [14–18]. Typically, the combined activities of the three kinds of effector proteins listed below decide the fate of a modified transcript: RNA modifying enzymes (writer proteins) that add a particular chemical group to an RNA molecule on a specific location; The reader proteins, known as RNA-binding proteins (RBPs), are able to recognize changed nucleotides, while the eraser proteins can take certain chemical groups off of modified nucleotides to restore them to their initial condition [19, 20].

A variety of RNA modification-related writers, readers, and erasers jointly determine their functional diversity [21–23]. For example, m^6^A modification is written under the catalyzation of m^6^A-methyltransferases complex, such as METTL3, METTL14, ZC3H13, RBM15, RBM15B, WTAP, and VIRMA. YTH domain-containing proteins, including YTHDC1, YTHDC2, and YTHDF3, then bind RNA in a m^6^A-dependent manner, so called m^6^A readers [23, 24]. Several investigations have demonstrated that YTHDF1 promotes m^6^A-modified RNA translation, YTHDF2 enables its degradation, and YTHDF3 boosts both roles. And all of them have been demonstrated to mediate phase separation [25–27]. Finally, several demethylases, such as FTO and ALKBH5, were identified to remove m^6^A modification from RNA [28, 29]. Increasing evidence indicates that RNA methyltransferases, RBPs, and demethylases are frequently overexpressed in human cancer tissue, leading to an increased expression of oncoproteins and onco-transcripts, and thus playing roles in cancer cell survival, proliferation, and metastasis. These proteins may be ideal targets for potential therapeutic interventions [30]. For example, FTO inhibitors have shown encouraging anticancer results in both *in vitro* and animal models of cancer [31, 32]. Recently, a number of experimental methods and computational tools have been developed to investigate the transcriptome-wide landscapes of various RNA modifications and their underlying regulatory roles [33–38]. However, the experimental technologies are usually laborious and costly, while the existing computational tools are aimed mainly for specific site prediction and have limitations in interpretability and generalizability as well as potential capacity limitation on discovering novel post-transcriptional regulations.

**Figure 1:**
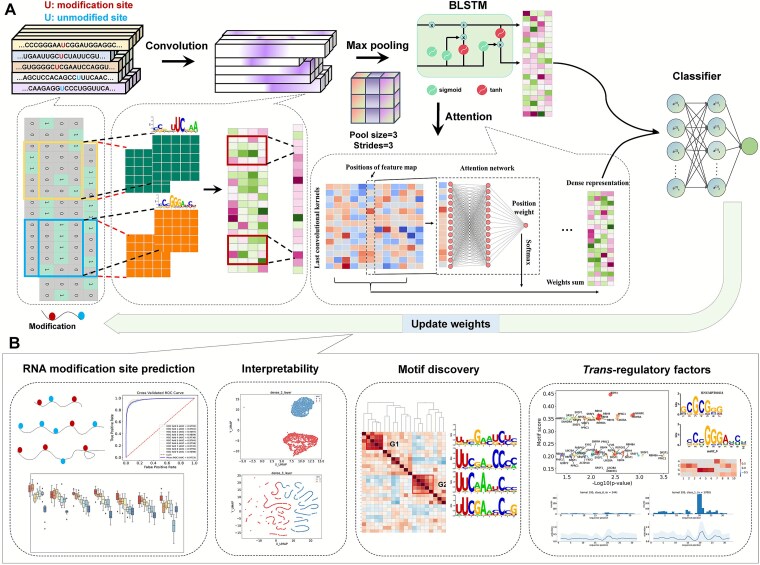
Overall workflow of model construction and application for NetRNApan. (A) Input data processing: Multiple RNA sequences (41-nt segments, 20-nt upstream, center nucleotide and 20-nt downstream) are one-hot encoded into a batch of 2D matrices (dimensions: 41 × 4) that serve as input to the model. Positive samples (modified sites) are shown in red, negative samples (unmodified sites) in blue. Network architecture: The model consists of: Convolutional layer with 256 filters (kernel size = 10, ReLU activation); max pooling layer (window size = 3, stride = 3); bidirectional LSTM layer (128 units each direction); attention mechanism layer; fully connected layers (256 units, ReLU activation); output layer (sigmoid activation). (B) Output applications: The model provides: Prediction scores (0–1 probability); motif discovery through convolutional filter visualization; RBP interaction analysis by motif comparison.

To tackle these challenges, in this study, we developed a deep neural network framework, namely NetRNApan, for RNA site prediction *de novo* from RNA nucleotides, motif discovery and trans-regulatory factors identification (Fig. 1). Specifically, using m^5^U profiles generated by FICC-seq and miCLIP-seq technologies [11], we first demonstrated the accuracy of NetRNApan in predicting potential RNA m^5^U modification sites. The overall performance of the NetRNApan resulted in area under curve (AUC) value of 0.97 by extensive computational evaluations, which outperformed 84 traditional machine-learning predictors and previous methods. Additionally, we visualized the contextual sequence features that NetRNApan captured and found that feature representation became more discriminative along the network layer hierarchy, suggesting that NetRNApan can effectively learn the intrinsic characteristics of m^5^U modification. More importantly, NetRNApan is not just a black box compared to traditional predictor in the prediction task; instead, it is easily interpretable. We found five representative motifs with consensus sequences that are crucial for m^5^U modification by clustering the informative motifs detected by NetRNApan. Furthermore, NetRNApan revealed promising m^5^U-associated RBPs. We found a total of 29 RBPs that had strong association with the detected motifs. Among them, ANKHD1, RBM4 and HNRNPK were previously reported to be involved in the regulatory mechanisms and function of RNA modification. Our further analyses of these RBPs provided new insights into post-transcriptional regulatory function related to m^5^U, such as gene expression, RNA splicing, and RNA transport. We further assessed the generalizability of NetRNApan by applying it to the prediction of m^6^A RNA modifications. A balanced and stringent training and validation dataset was constructed based on prior studies and available data. We then extensively evaluated the model’s performance, achieving an AUC of 0.87 on the validation set—surpassing the performance of existing tools. Furthermore, leveraging the interpretability of NetRNApan, we identified potential interactions between RBPs and m^6^A modifications that do not depend on the canonical RRACH motif. Among which, YBX3 and XRN2 has been reported to be interacting with m^6^A modification [1, 39, 40]. In summary, NetRNApan is a robust and interpretable deep learning framework, which can be useful to explore and understand the underlying regulatory mechanisms of RNA modification. NetRNApan is freely available at https://github.com/bsml320/NetRNApan.

## Materials and methods

### Data collection and feature encoding

For training and validation of m^6^A predictor, we obtained 18 977 base-resolution m^6^A sites (HighRes) and 2 120 434 low resolution m^6^A peaks (LowRes) in human from m6A-Atlas (v2.0) [39]. To construct negative dataset, we acquired human genome annotation and sequences from GENCODE (GRCh38p14) [41]. For the development of a high-quality training and testing dataset, we selected 14,855 HighRes sites located within exonic regions as positive data. Additionally, we extracted all RRACH sequences (where R represents A/G and H denotes A/C/U) from human exons to serve as candidate negative sites. Subsequently, we eliminated candidate negative sites that fell within the range of m^6^A peaks (LowRes) to mitigate the inclusion of potential positive sites within the negative dataset. To ensure genomic distribution consistency between positive and negative datasets, we computed the ratios of positive sites in the 5' UTR, CDS, and 3' UTR regions and adjusted the negative dataset by random selection to match these ratios. Ultimately, we utilized the entire set of positive data and randomly selected 74 275 negative sites to construct the training and testing dataset, maintaining a positive-to-negative ratio of 1:5. We employed 80% of this dataset for training the NetRNApan and DeepM6ASeq models [42], when the remaining 20% was used for the test set (2971 positive and 14,855 negative sites without cell line specificity). For the evaluation of DeepM6ASeq and SRAMP, we employed 41-nucleotide (nt) segments to represent m^6^A sites, while a segment length of 101 nt was used for evaluating CLSM6A.

To facilitate fair comparison for prediction of m^5^U modification, the training and testing datasets were obtained and processed from the previous study [43] which was deposited at NCBI Gene Expression Omnibus (GEO, accession ID: GSE109183). This dataset was generated by miCLIP-seq and FICC-seq on two cell lines (HAP1 and HEK293) [11] and contained 3696 experimentally identified m^5^U sites. To make the most of training data, we did not apply any additional filter for the positive dataset. To capture the surrounding sequence characteristics, each m^5^U site was represented by an RNA segment that was 41 nucleotide (nt) long, with flanking 20 nt upstream and downstream of the m^5^U site, respectively. To create balanced datasets, we randomly chose unmodified Us from genome as negative data and matched the size to positive dataset as previous study described [43, 44]. The created dataset was randomly separated into two subsets: training data (90% of total data) and independent data (10% of total data, 369 positive and 370 negative sites without cell line specificity). Furthermore, for the benchmarking comparison, a total of 12 types of characteristics based on sequence and physicochemical properties were constructed and analyzed.

### Composition of K-spaced nucleic acid pairs

Here, we defined the **R**NA **M**odification **N**ucleotide, abbreviated as RMN (20, 20), to represent each modification site with 20 nucleotides upstream and 20 nucleotides downstream. The composition of K-spaced nucleic acid pairs (CKSNAP) feature encoding is made up of nucleotide pairs that are *k*-steps apart in a RMN (20, 20). We specifically calculated the probability of a nucleotide pair with two nucleotides at places *a* and *a* + *K* + 1, where *a* = 1, …, (*l*-*K*-1) and *l* = 41. The nucleotide GU, for instance, with K = 4 steps away, illustrates the following case: $AC\underset{\_}{G}\underset{K}{\underbrace{GUAC}}\underset{\_}{U} UACGU$, where G and U are located at the position *a* = 3 and *a* = 8, respectively. For *K* = 0, a feature vector is computed as shown below.


\begin{align*} {\left(\frac{N_{AA}}{N_{Total}},\frac{N_{AC}}{N_{Total}},\frac{N_{AG}}{N_{Total}},\frac{N_{AU}}{N_{Total}}, \dots, \frac{N_{UU}}{N_{Total}}\right)}_{K=0},\\{N}_{Total}={N}_{AA}+{N}_{AC}+\dots, {N}_{UU} \end{align*}

### Tri-nucleotide composition

The continuous tri-nucleotide pair composition of the RMN (20, 20) is determined by the Tri-nucleotide composition (TNC) feature encoding. The TNC encoding has a total of 64 descriptors:


$$ D\left(i,j,k\right)=\frac{N_{(ijk)}}{N-2},i,j,k\in \left\{A,C,G,U\right\} $$

where *N_(ijk)_* donates the number of tri-nucleotides represented by nucleotide types *i*, *j* and *k*.

### Pseudo K-tuple nucleotide composition and pseudo dinucleotide composition

Local and global sequence-order information can be incorporated via the pseudo K-tuple nucleotide composition (PseKNC) feature, while pseudo dinucleotide composition (PseDNC) is a special case of PseKNC, in which K equals to two, here we use PseDNC as example to show how PseKNC is calculated [45]. Following is a definition of the PseDNC encoding:


$$ P={\left({p}_1,{p}_2,\dots, {p}_{16},{p}_{16+1},\dots, {p}_{16+\lambda }\ \right)}^T $$

$$ {p}_k=\left\{\!\!\begin{array}{c}\frac{f_k}{\sum_{i=1}^{16}{f}_i+w\sum_{j=1}^{\lambda }{\theta}_j},\kern0.5em \left(1\le k\le 16\right)\\[10pt] {}\frac{w{\theta}_{k-16}}{\sum_{i=1}^{16}{f}_i+w\sum_{j=1}^{\lambda }{\theta}_j},\kern0.5em \left(17\le k\le 16+\lambda \right)\end{array}\right. $$

where *f_k_* (*k* = 1, 2, …, 16) represents a dinucleotide’s normalized frequency in the RMN (20, 20), λ indicates the highest produced rank of the correlation along the RMN (20, 20), the weight factor is represented by *w* (0 to 1), and *θ_j_* (*j* = 1, 2, …, *λ*) is the *j*-tier correlation factor, which is defined as:


$$ {\theta}_1=\frac{1}{L-2}{\sum}_{i=1}^{L-2}\varTheta \left({R}_i{R}_{i+1},{R}_{i+1}{R}_{i+2}\ \right) $$

$$ {\theta}_{\lambda }=\frac{1}{L-1-\lambda}\sum_{i=1}^{L-1-\lambda}\varTheta \left({R}_i{R}_{i+1},{R}_{i+\lambda }{R}_{i+\lambda +1}\ \right) $$

where the correlation function is defined:


$$ \varTheta \left({R}_i{R}_{i+1},{R}_j{R}_{j+1}\ \right)=\frac{1}{u}\sum_{u=1}^u{\left({C}_u\left({R}_i{R}_{i+1}\right)-{C}_u\left({R}_j{R}_{j+1}\right)\right)}^2 $$

where the number of physicochemical indices is indicated by *μ*. The six physicochemical variables examined in this study were rise, roll, shift, slide, tilt and twist. C*_u_* (R*_i_* R*_i + 1_*) is the numerical representation of the *u*-*th* physicochemical index of the dinucleotide R*_i_*R*_i + 1_* at position *i*, while C*_u_* (R*_j_*R*_j + 1_*) stands for the dinucleotide (R*_j_*R*_j + 1_*) 's matching value at position *j*.

### Nucleotide chemical property

Unlike one-hot encoding which represents categorical nucleotide identity, nucleotide chemical property (NCP) encoding captures continuous chemical properties that are shared among nucleotides, providing a complementary feature space that may reveal different aspects of sequence-function relationships. Specifically, all nucleotides are split into three main types, each of which has a different chemical structure and binding property, according to their chemical characteristics:


$$ \mathrm{Ring}\ \mathrm{Structure}=\left\{\!\!\begin{array}{c} Purine,\kern0.5em \mathrm{A},\mathrm{G}\kern1.25em \\{} Pyrimidine,\kern0.5em \mathrm{C},\mathrm{U}\kern0.5em \end{array}\right. $$

$$ \mathrm{Functional}\ \mathrm{Group}=\left\{\!\!\begin{array}{c} Amino,\kern0.5em \mathrm{A},\mathrm{C}\kern1em \\{} Keto,\kern0.5em \mathrm{G},\mathrm{U}\kern0.5em \end{array}\right. $$

$$ \mathrm{Hydrogen}\ \mathrm{Bond}=\left\{\!\!\begin{array}{c} Weak,\kern0.5em \mathrm{A},\mathrm{U}\ \\{} Strong,\kern0.5em \mathrm{C},\mathrm{G}\kern1em \end{array}\right. $$

The *i*^th^ nucleotide in a RMN (20, 20) is identified using the following formulae, which incorporates these chemical characteristics [46, 47]:

${R}_i=\left\{\!\!\begin{array}{c}1,\kern0.5em \mathrm{if}\ {N}_i\in \left\{A,G\right\}\\{}0,\kern0.5em \mathrm{if}\ {N}_i\in \left\{C,U\right\}\end{array}\right.$ ${F}_i=\left\{\!\!\begin{array}{c}1,\kern0.5em \mathrm{if}\ {N}_i\in \left\{A,C\right\}\\{}0,\kern0.5em \mathrm{if}\ {N}_i\in \left\{G,U\right\}\end{array}\right.$  ${H}_i=\left\{\!\!\!\begin{array}{c}1,\kern0.5em \mathrm{if}\ {N}_i\in \left\{A,U\right\}\\{}0,\kern0.5em \mathrm{if}\ {N}_i\in \left\{C,G.\right\}\end{array}\right.$

### One-hot encoding

The one-hot encoding [47–49], which provides the position-specific nucleotide composition of the DNA/RNA sequence, is frequently employed in sequence-based prediction applications. This method produces a sparse one-hot encoding representation where each nucleotide is uniquely encoded by a 4-dimensional vector containing a single ‘1’ (representing the specific nucleotide type) and three ‘0’ (representing its absence).​ Specifically, A is specifically encoded by (1, 0, 0, 0), C by (0, 1, 0, 0), G by (0, 0, 1, 0), and U by (0, 0, 0, 1), in that order. A digital vector for an RMN (20, 20) is represented as:


$$ P=\left({p}_{n1},{p}_{n2},{p}_{n3},{p}_{n4},\dots, {p}_{n41}\ \right),p\in \left\{\!\!\!\begin{array}{c}\mathrm{A}:1,0,0,0\\{}\mathrm{C}:0,1,0,0\\{}\mathrm{G}:0,0,1,0\\{}\mathrm{U}:0,0,0,1\end{array}\right.,n\in \left\{A,C,G,U\right\} $$

### Accumulated nucleotide frequency

The nucleotide density and the distribution of an RNA nucleotide is referred to as accumulated nucleotide frequency (ANF) feature coding [50, 51]. The following formula is used for calculating the ANF for every position in RMN (20, 20), which results in a 4-dimensional vector representing the cumulative frequencies of A, C, G, and U:


$$ {d}_l=\frac{1}{l}\sum_{j=1}^lf\left({n}_j\right),\kern1.75em f\left({n}_j\right)=\left\{\!\!\!\begin{array}{c}1,\kern0.5em if\ {n}_j=q\\{}0,\kern0.5em other\end{array}\right.,l=1,\dots, 41 $$

where ${n}_j$ represents the nucleotide at the *j*^th^ position and $q\in \left(\mathrm{A},\mathrm{C},\mathrm{G},\mathrm{U}\right)$. Taking the RNA sequence of ‘GAUAGGUG’ as an example. When *l* = 4 (the subsequence ‘GAUA’), the nucleotide at the *l*^th^ position is A and the density of this position is calculated as ${d}_4=\frac{1}{4}\times \sum_{j=1}^4f\left({n}_j\right)=\frac{1}{4}\times \left[0+1+0+1\right]=0.5$. Therefore, the ANF vector for this position is calculated as [0.5(A),0(C),0.25(G),0.25(U)]. This calculation was made for the density of all the 41 positions, resulting in a feature vector of length 41 × 4 = 164 for each RNA segment.

### Other sequence features

A series of features about the nucleic acid composition were described in the supplementary material. The following additional feature encoding schemes were also constructed: nucleic acid composition (NAC) feature encoding reflects the nucleotides frequencies of the sequence fragments surrounding the modification site (RMN (20,20)); di-nucleotide composition (DNC) feature encoding represents the composition of the composition of continuous di-nucleotide pairs in the RMN (20, 20); tri-nucleotide composition (TNC) feature encoding represents the composition of the composition of continuous tri-nucleotide pairs in the RMN (20, 20); enhanced nucleic acid composition (ENAC) feature encoding calculates the local NAC based on the sequence window of fixed length (the window was set as five in this study) that continuously slides from the 5′ to 3′ terminus of each nucleotide sequence and can be usually applied to encode the nucleotide sequence with an equal length; Composition of K-spaced Nucleic Acid Pairs (CKSNAP) feature encoding represents the composition of nucleotide pairs that are separated by K (K = 0, 1, 2, …, 5. The default maximum value of K is 5 in this study) other nucleotide within the RMN (20, 20); Kmer encoding calculates the occurrence frequencies of k neighboring nucleotide in the RMN (20, 20); Series Correlation Pseudo Dinucleotide Composition (SCPseDNC) reflects a series of correlation pseudo dinucleotide composition in the RMN (20, 20).

### NetRNApan construction

Figure 1 illustrates the implementation of a new learning framework called NetRNApan to predict and analyze RNA modification sites using nucleotides as input. A number of computing units termed neurons were included in each layer, serving as an internal feature representation. One-hot encoding was used to transform the modified nucleotides into a binary matrix (*L* × 4), where *L* stands for the length of the nucleotides (41 nt: 20 nt upstream +1 center nucleotide +20 nt downstream). The 4 columns represent the one-hot encoding vectors for the four nucleotide types: A: [1,0,0,0], C: [0,1,0,0], G: [0,0,1,0], U: [0,0,0,1]. The binary matrix was then input into a convolution layer to find nucleotides sub-motifs. Multiple kernels serve as the essential elements in the convolution layer, which was frequently employed for motif identification [52–55]. From the input nucleotides, typical patterns were initially found via a large number of convolution kernels in the NetRNApan. To determine the maximum activation regions over globally contiguous regions and subsequently to summarize the most active pattern in the sequences, a maxpooling layer was used after the convolutional layer. ​This max pooling operation was applied along the sequence dimension with a window size of 3 nucleotides and a stride of 3. Max pooling’s down sampling method reduces the ​sequence length, which increases the adaptability of the deep learning model.

Next, we included a BLSTM layer in NetRNApan to further extract the broad interdependence of long-range sequence among identified patterns from both forward and backward directions. The inclusion of a BLSTM is justified by the possibility that many evenly spaced amino acids may control how chemical group binds to the nucleotide. The ability of BLSTM makes it easier for the model to capture combinations of nucleotides or relationships among nucleotides at various places. The BLSTM unit consists of four components: three gates (input, forget and output), and a single cell that remembers characteristics across arbitrary intervals. In particular, taking the input with length *L* as ${\left\{{x}_p\right\}}_{p=1}^L$in BLSTM, and for each location *p*, assigns the input gate to *I_p_*, forget gate to *F_p_*, output gate to *O_p_*, hidden state to *H_p_*, and cell state to *C_p_*. The following is the BLSTM training procedure:


$$ {F}_p=\sigma \left({W}_f\times \left[{x}_p,{H}_p-1\right]+{b}_p\right) $$

$$ {I}_p=\sigma \left({W}_I\times \left[{x}_p,{h}_p-1\right]+{b}_I\right) $$

$$ {C}_p={F}_p\times{C}_{p-1}-{I}_P\times \mathit{\tanh}\left({W}_C\times \left[{x}_p,{h}_p-1\right]+{b}_C\right) $$

$$ {O}_p=\sigma \left({W}_O\times \left[{x}_p,{h}_p-1\right]+{b}_O\right) $$

$$ {H}_p={O}_p\times \mathit{\tanh}\left({C}_p\right) $$

A layer called attention was introduced after the BLSTM layer in NetRNApan, aiming to identify the most prevalent sequence patterns. By conquering every BLSTM layer hidden state and assigning the important locus with larger weight, the attention layer helped to find more useful features [56]. Theoretically, the attention layer produces the output vector *A* as illustrated below by using the hidden variables ${\left\{{B}_P\right\}}_{p=1}^L$taken from the BLSTM layer as input.


$$ {\alpha}_p=\frac{\exp \left(w\left({B}_p\right)\right)}{\sum_{i=1}^L\exp \left(w\left({B}_i\right)\right)} $$

$$ A=\sum_{p=1}^L{\alpha}_p{B}_p $$

where *w* is an illustration of a neural network calculating a scalar weight. Then, the variables generated by the attention layer were combined with a fully linked layer to reveal the nonlinear relationships. One sigmoid neuron in the final output layer calculates a score for the provided input *y*, where *S_m_* is defined as


$$ {S}_m(y)= sigmoid(y)=\frac{1}{1+{e}^{-y}} $$

The *S_5_* value, which is within the range from 0 to 1, represents the likelihood that an input is a real RNA modification site, which ranges from 0 to 1.

### Model training and evaluation

The NetRNApan was trained using the Adam optimizer and the mini-batch method. Binary cross-entropy was employed to measure the difference between the target and predicted labels. We then trained the deep learning network to reduce this loss. Each training session was followed by a test against the validation dataset, and the related loss and accuracy scores were collected. To prevent model overfitting, we incorporated an early stop mechanism during training. The model was continuously improved until the validation accuracy did not continue to increase for 20 iterations. To assess the reliability and accuracy of the NetRNApan, 10-fold cross-validation (CV) and independent testing were carried out. Several measures, including accuracy (Acc), specificity (Sp), sensitivity (Sn), and the area under the receiver operating characteristic (ROC) curve (AUC), were calculated. To obtain the greatest performance, the Hyperopt tool was used, and a range of parameters, including the kernel size, filter numbers, mini-batch size, learning rate, and dropout probability, were tuned. Hyperopt searches the given hyperparameter space by a Bayesian mechanism. Specifically, separate training (inner loop) and validation (outer loop) sets were used to conduct 100 assessments in in a process similar to nested cross-validation. The related AUC values were generated for each set of parameters. The best parameters were picked from the collection of parameters with the highest AUC values. All the training processes were performed using four Tesla V100. The Keras (version 2.3) and the tensorflow-gpu (version 1.15) were adopted.

### Conventional machine learning (ML) classifiers

We used seven algorithms to implement 84 traditional machine-learning classifiers for predicting RNA modification sites based on 12 features: AdaBoost (AB), decision trees (DT), gradient boosting classifier (GBC), k-nearest neighbors (KNN), logistic regression (LR), random forests (RF), and stochastic gradient descent (SGD). The five-fold cross-validation (CV) was used to evaluate each predictor’s prediction capacity. The ROC curves for *Sn* versus *1-Sp* scores were drawn, and the AUC values were determined. To evaluate performance reliably, the five-fold CV was run 10 times individually, and the average AUC values for each model parameter were determined. To find the best settings, we explored dozens to hundreds of different parameter combinations for each model.

### Analysis of motif extracted from filters within the convolution layers

As demonstrated in DeepBind [53] and Basset [57], filters within the layer of convolution are strong motif detectors. We retrieved and displayed the sequence motifs detected by NetRNApan to interpret our model. Specifically, through identifying all subsequences that activate the convolutional filters after scanning the whole input sequence with m^5^U sites, we generated the positional weight matrix (PWM) for each motif. In specifically, we can obtain the total number of outputs *N* (calculated as follows) for each filter with an input sequence of length *L*, a sliding window of size *T*, and a stride of 1:


$$ N=L-T+1 $$

Suppose that an output value is indicated by O*_i_*, the matching subsequence C*_i_* can be derived as:


$$ {C}_i=Q\left[i:i+T\right] $$

where *Q* represents the input sequence. If O*_i_* is above the average score, C*_i_* is extracted to create the PWM. The motif score M_s_ was calculated by subtracting the maximal activation values for the positive and negative classes, which determines a motif enrichment for the positive class and correlates to how crucial the kernel is for deciding whether a sequence is m^5^U or not.


$$ {M}_s= Aver\left({S}_p\right)- Aver\left({S}_n\right) $$

where *S_p_* and *S_n_* stand for the average activation scores in positive and negative class.

### Identification of trans-regulation of m^6^A beyond RRACH

To identify potential RBPs that interact with m^6^A modification sites independently of the RRACH motif, we first acquired RBP peak data from 250 eCLIP experiments available through ENCODE [58]. RBP motifs were detected using HOMER [59] with the parameters ‘-rna -len 10 -p 20 -S 10.’ The top five motifs identified from each eCLIP experiment were converted to MEME format and subsequently compared to position weight matrices (PWMs) extracted from the convolutional layers of NetRNApan using TOMTOM [60].

### Identification of functional RNA-binding protein in regulation of m^5^U

To further uncover the prevalent sequence patterns that are critical for RNA modification, all PWMs were clustered using hierarchical clustering method according to Spearman correlations among the nucleic acid composition of PWMs that were detected. Additionally, NetRNApan could generate a protein-binding perspective for exploring the function of RNA modifications. We compared the motifs learned by NetRNApan with the known RBP motifs. RBPs whose binding site favor was shown to be strongly linked with the derived motifs were uncovered. Functional enrichment analysis of these identified RBPs was performed to further explore the potential regulatory roles of RNA modification.

## Results

### NetRNApan accurately predicts RNA m^5^U modification

**Figure 2:**
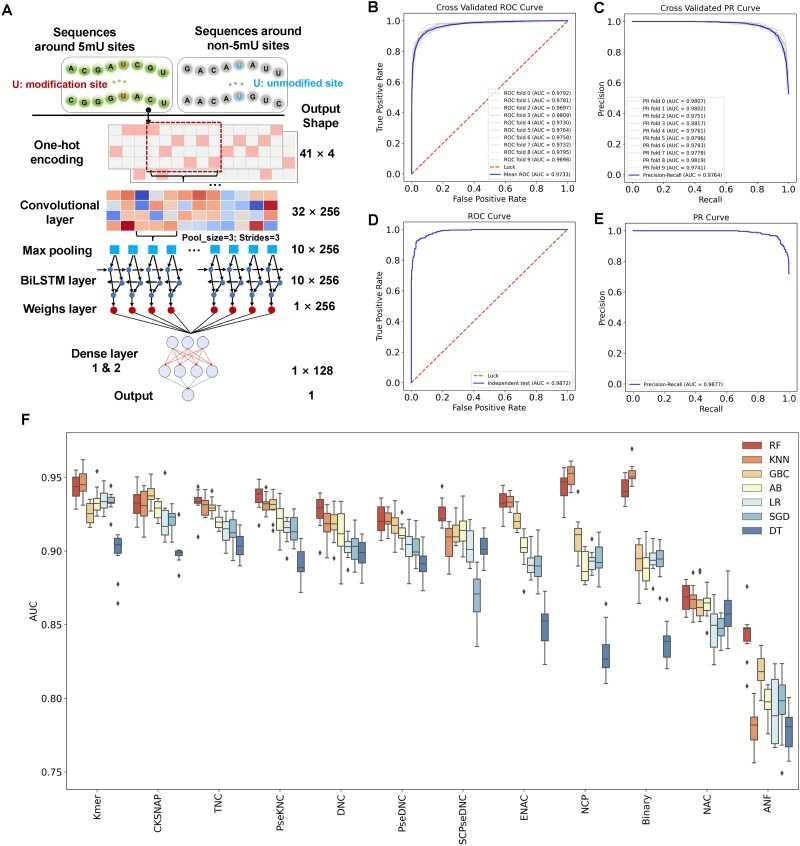
Performance and evaluation for NetRNApan. (A) The implementation of NetRNApan to predict and analyze RNA modification utilizing m^5^U profiles as a case. (B, C) The area under the receiver operating characteristic (ROC) curve (AUC) and precision-recall (PR) curve values for NetRNApan were obtained with 10-fold cross-validation (CV). (D, E) ROC curves, PR curves and the AUC values for NetRNApan with the independent test dataset. F. Performances of 84 traditional machine-learning models for the 12 types of features. 5-fold CV was used for determining the AUC values.

Recent research has shown that deep learning is powerful for mining sequence information, images analysis, and natural language processing. In this study, a deep learning-based framework, namely NetRNApan, was developed for RNA modification prediction and analysis. The implementation of NetRNApan consists of five components: the input layer, convolution-pooling modules, BLSTM layer, attention layer and the output layer, which automatically learns informative classification features. We take m^5^U modification as an example to illustrate the powerful capacity of our method in many tasks, including RNA site prediction, motif discovery and trans-regulatory factors identification (Fig. 2A). First, the 10-fold CV was executed to assess NetRNApan’s prediction performance on the training dataset. The ROC curves were drawn, and the corresponding AUC values were calculated. NetRNApan performed well, with an average AUC value of 0.9733 by 10-fold CV, ranging from 0.9697 to 0.9809 (Fig. 2B), suggesting its good and reliable predictive power. NetRNApan prediction robustness was evaluated as well using the area under the precision-recall (AUPR) curve. The PR curve represents the trade-off between the number of false positive predictions and the number of false negative predictions. NetRNApan achieved AUPR values ranging from 0.9741 to 0.9819 during the balanced sample with 10-fold CV training (Fig. 2C), indicating that our model had great potential in predicting true positive sites with the high precision. Furthermore, we examined the generalizability of our models using an independent dataset that was not part of the training set. NetRNApan had the AUC value and AUPR of 0.9872 and 0.9877, respectively (Fig. 2D and E). These good and consistent metrics between 10-fold CV and independent testing indicated the promising accuracy and robustness. In addition, in our comparison of NetRNApan with other existing tools using an independent dataset, NetRNApan had AUC value improvement by over 3.5% (0.987 *versus* 0.954) for the modification sites prediction when compared to m5UPred [43].

### NetRNApan outperforms traditional machine-learning predictors and is easily interpretable

**Figure 3:**
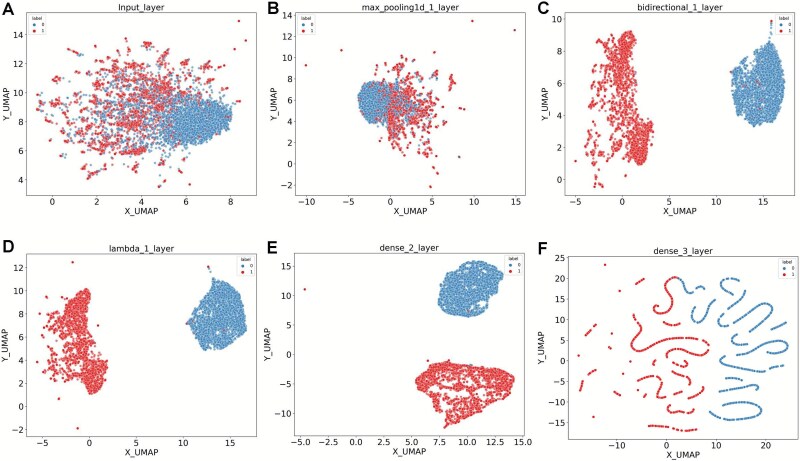
The capacity of NetRNApan for feature representation along the network layer hierarchy in the training dataset. (A) Feature representation of modified and unmodified nucleotides in the NetRNApan input layer applying UMAP method. (B) Feature representation of the modified and unmodified nucleotides in the CNN layer. (C) Feature representation of the modified and unmodified nucleotides in the BLSTM layer. (D) Feature representation of the modified and unmodified nucleotides in the attention layer. (E) Feature representation of the modified and unmodified nucleotides in the fully connected layer. (F) Feature representation of the modified and unmodified nucleotides in the final output layer. UMAP, uniform manifold approximation and projection; CNN, convolutional neural network; BLSTM, bidirectional long short-term memory.

**Figure 4:**
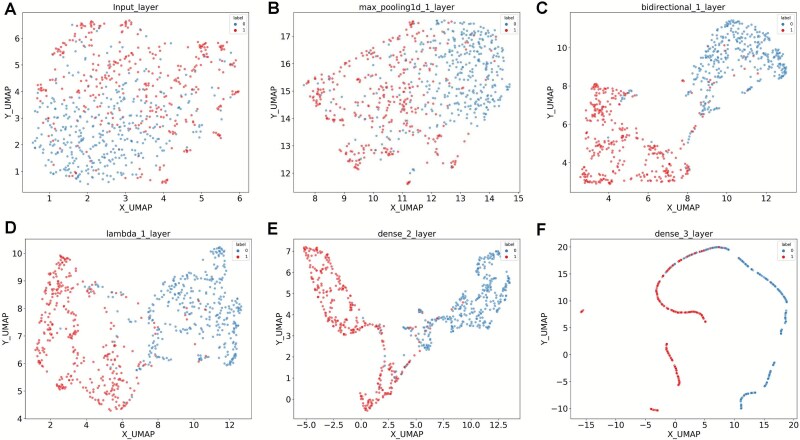
Feature representation of NetRNApan in different layers using the independent test dataset. (A) Nucleotides feature representation for modified and unmodified sites in the NetRNApan input layer. (B) Nucleotides feature representation for modified and unmodified sites in the CNN layer. (C) Nucleotides feature representation for modified and unmodified sites in the BLSTM layer. (D) Nucleotides feature representation for modified and unmodified sites in the attention layer. (E) Nucleotides feature representation for modified and unmodified sites in the fully connected layer. (F) Nucleotides feature representation for modified and unmodified sites in the final output layer.

For performance comparison and benchmarking, we encoded 12 widely used features in RNA modification predictions, including nine sequence-based features (ANF, Binary, CKSNAP, DNC, ENAC, Kmer, NAC, TNC, and RCKmer) and three types of physicochemical properties-based features (EIIP, NCP, and PseDNC) (Supplementary Table S1). 84 conventional machine-learning predictors were developed based on 12 types of features with seven algorithms (SVM, RF, LR, AB, SGD, DT, KNN and GB) (Fig. 2F). The average AUC value of individual predictor was calculated based on 10-fold CV. It should be noted that parameters of all predictors have been extensively tuned. Although the performance of different predictors varies, our results showed all of them have predictive power (with AUC value over 0.5). The results revealed random forest (RF) had the best performance, having an average AUC value of 0.92 across 12 types of features. In addition, serval features such as Kmer, CKSNAP and TNC, performed well, achieving the average AUC values > 0.90 (Fig. S1). In comparison, we found that the performance of NetRNApan was better than other 84 conventional machine-learning predictors, resulting in the AUC value improvements from 3.66% to 26.94%. The accuracy and robustness of the NetRNApan might be partly attributed to its deep neural network architecture, which is easily interpretable compared to the traditional machine-learning algorithms. The inputs can be projected via the hidden layers of NetRNApan to a representation space with lower dimensions. We used the UMAP approach to visually display the m^5^U sites and non-m^5^U sites in the training dataset based on the feature learnt at various network layers to demonstrate the capabilities of hierarchical representation using NetRNApan. We found that the feature representation became more discriminative along the network layer hierarchy (Fig. 3). More specifically, the feature representations for m^5^U sites and non-m^5^U sites were mixed at the input layer. As the model continued to train, all nucleotides were grouped into two distinct clusters by the low-dimensional projection, reflecting binding specificities between m^5^U sites and non-m^5^U sites. Similarly, the powerful generalization ability of NetRNApan is also verified in independent test sets, resulting in two distinct clusters with clear boundary for m^5^U sites and non-m^5^U sites (Fig. 4).

### NetRNApan identifies consensus motifs potentially important for RNA modification

**Figure 5:**
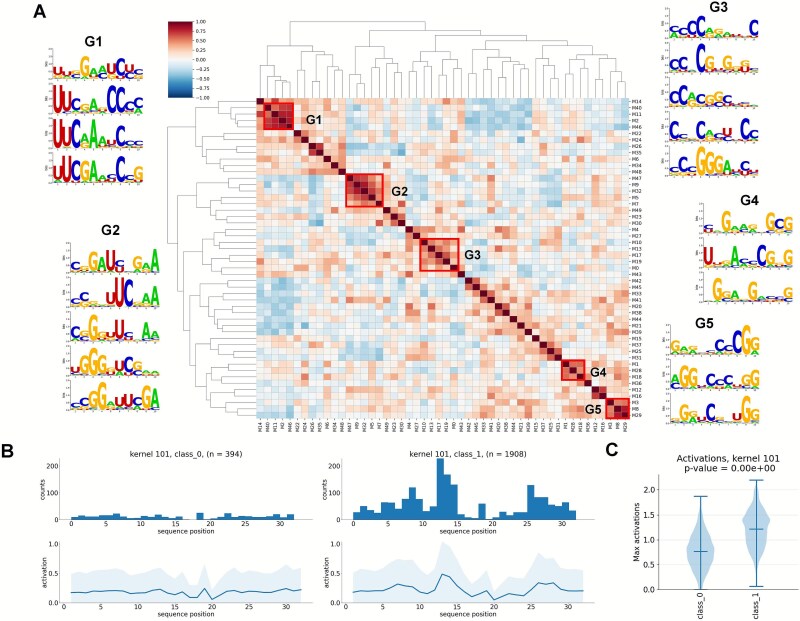
Consensus motifs revealed by NetRNApan. (A) Five consensus motif clusters were revealed by hierarchical clustering of informative features that were extracted from the most active kernels in the NetRNApan convolutional layer. (B) Histograms and line charts showed the maximum activation positions where the motif (Kernel 101) was extracted. (C) Violin plot showed the distribution of the maximum activation scores for motif (Kernel 101) among modified and unmodified nucleotides.

To interpret our deep learning model, we also decoded and analyzed the sequence features captured by our model from input nucleotides. Briefly, we first obtained the local segments captured by 256 convolution filters in the first convolution layer of our model. Each filter with a length of 10 nucleotides that was maximally activated at different regions of input. These activated features were then overlaid together to create position weight matrices (PWMs), which were considered as local motifs. To determine the representative motifs, motif score was calculated to measure the difference in the mean maximum activation scores between positive class and negative class. In total, 135 informative motifs were identified. Some strong motifs with higher scores were significantly enriched in positive samples such as ‘xCxGGG[A/U]x[C/U]U’ (Kernel 101, score = 0.446, *P* < .01), ‘[C/U]xGxxxxGCG’ (Kernel 138, score = 0.379, *P* < .01), and ‘UUCGAxxCxG’ (Kernel 195, score = 0.365, *P* < .01). All motifs and the corresponding PWMs were graphically illustrated (Figs S2–S4). Due to the significance of some motifs in modification, they could be found more than once. Then, the top 50 motifs were subjected to clustering analysis for further pattern mining. The pairwise Spearman’s correlations between PWM of each motif were evaluated and hierarchical clustering was performed to the correlation matrix, yielding five representative motif clusters (Fig. 5A), such as UUCGAx[U/C], [C/G]GGUU[C/U]xAA, GGxCCCGG. We further analyzed one of the top-scoring motifs (Kernel 101, score = 0.446), and displayed the activation positions and distribution of the activation scores between m^5^U and non-m^5^U nucleotides (Fig. 5B and C). It was found that the motif had significantly greater activation scores and was enriched in modification sites and surrounding locations. Taken together, NetRNApan could identify particular patterns of recognition and revealed consensus motifs that may be significant for RNA modifications.

### NetRNApan provides new insights into post-transcriptional regulation

**Figure 6:**
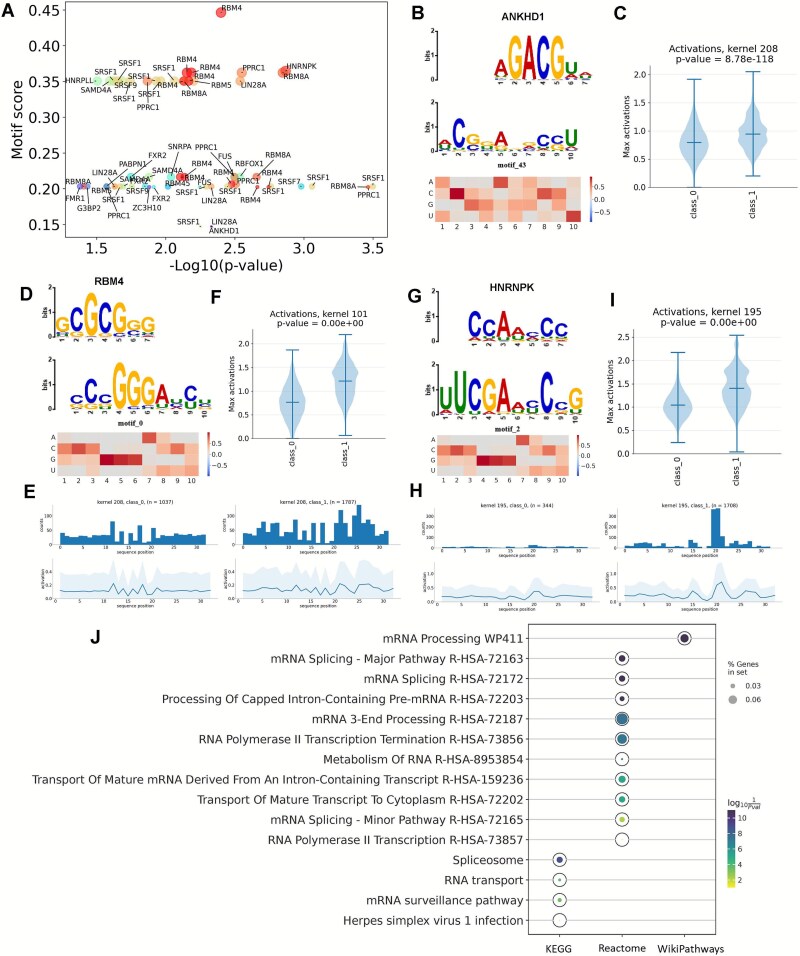
NetRNApan decodes trans-regulatory factors form a protein-binding perspective. (A) The top 50 scoring-motifs derived from NetRNApan were found to correspond with a total of 29 RNA-binding proteins (RBPs) with statistical significance. The statistical significance cutoff was set as *P* < .01. (B–I) Graphic logo, position weight matrix (PWM) and maximum activation distribution for Kernel 208 (B, C), Kernel 101 (D–F) and Kernel 195 (G–I) extracted from NetRNApan, which matches three RBPs, ANKHD1, RBM4, and HNRNPK, respectively. J. The pathway enrichment analysis of all identified RBPs based on three gene annotation sets (KEGG, Reactome, and WikiPathways).

There is growing evidence that the RNA epigenetic dysfunction contributes to the development of human disease, especially cancers [14]. Writers, erasers, and readers of different RNA modifications are all involved in these dysfunctions, with each kind of these effector proteins having a specific role in disease [61]. Compared with the well characterized m^6^A modification and its increasingly prominent regulatory mechanism, there are still many unknown types of RNA modification. NetRNApan can provide a protein-binding perspective for understanding the functions of RNA modifications. In this study, we discovered 21 RBPs whose binding sites were significantly linked to the extracted motifs among the top 50 top-scoring motifs (Fig. 6A, Supplementary Table S2). For example, among all the identified RBPs, we found that the motif-43 identified by NetRNApan could be matched to the binding motif of ANKHD1 (*P*-value: 4.68E-03) (Fig. 6B and C), which was found to interact with the major m^5^U methyltransferase TRMT2A [62]. ANKHD1 is a large protein characterized by the presence of multiple ankyrin repeats and a K-homology (KH) domain, and its KH domain binds to RNA or ssDNA and is associated with transcriptional and translational regulation [63]. In addition, we found that the ‘CGCGGG’ motif identified by NetRNApan could be matched to the binding motif of RBM4 (*P*-value: 4.68E-03) (Fig. 6D–F). RBM4 is a splicing inhibitor that acts as a tumor suppressor by regulating cancer-related splicing [64]. RBM4 was shown to interact with YTHDF2 and to be a potential inhibitor of M1 macrophage polarization through the degradation of m^6^A-modified STAT1 mRNA [65]. Additionally, the ‘UUCGAxxCxG’ pattern discovered by NetRNApan matches the binding motif of HNRNPK (*P*-value: 1.42E-03) (Figs 6G–I). Li *et al.* revealed that by identifying the splice site (TCCCTA) of the RBM4 pre-mRNA, HNRNPK, serving as another pair of scissors, directly promotes RBM4 exon skipping, increasing the production of the tumor-promoting isoform RBM4-S [66]. Our prediction results may help generate further hypothesis on crosstalk of different RNA modifications. In addition, functional enrichment analysis of these identified RBPs was also performed to further explore the potential regulatory roles of m^5^U (Fig. 6J, Fig. S5). We found that several terms, including G-quadruplex RNA binding, regulation of (alternative) mRNA splicing, RNA transport and gene expression were enriched, which implied for their potential critical roles in transcriptional regulation (Supplementary Table S3, S4). Though additional experiments are required to validate these findings, our deep learning framework and prediction results may facilitate the discovery of underlying regulation mechanisms of RNA modifications.

### NetRNApan accurately predicts m^6^A modifications and reveals hidden regulation

**Figure 7:**
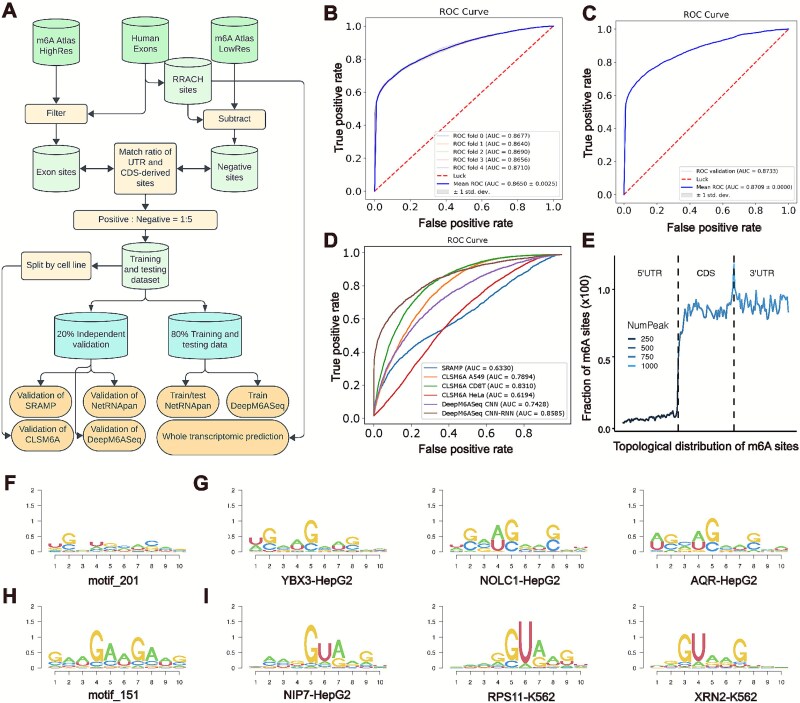
NetRNApan accurately and robustly predicts m^6^A modification and reveals potential regulating RBPs independently of the RRACH motif. (A) Workflow of constructing training and testing dataset for m^6^A prediction of NetRNApan. (B) The area under the receiver operating characteristic (ROC) curve (AUC) of NetRNApan obtained from 5-fold cross-validation. (C) ROC and AUC of NetRNApan on independent testing dataset. (D) ROC and AUC of existing tools on prediction of m^6^A modification. (E) Topological distribution of predicted m^6^A sites along transcripts. (F–I) Sequence pattern extracted from convolutional layer Kernel 201 (F) and Kernel 151 (H) of NetRNApan. As well as sequence motif of RBPs showing high similarity to motif_201 (G) and motif_151 (I).

To evaluate the generalizability of NetRNApan across different RNA modification types, we tested and validated our model specifically for m^6^A modifications. For comparative performance analysis, we selected three representative tools: SRAMP [67], due to its high citation count; DeepM6ASeq [42], for its provision of user-specific training options; and CLSM6A [68].given its recent publication. We initially constructed a well-defined training and testing dataset by integrating both high-resolution (base resolution) and low-resolution (peak resolution) m^6^A sites/peaks (Fig. 7A). To minimize the risk of incorrectly labeling unidentified m^6^A sites as negative, we first extracted exon sequences matching the RRACH pattern (R: A/G, H: A/C/U) and excluded those overlapping with known m^6^A peaks (Fig. 7A). Additionally, we ensured a balanced representation by matching the ratios of sites in the 5′ UTR, CDS, and 3′ UTR regions between positive and negative datasets, and by maintaining a positive-to-negative site ratio of 1:5. This approach resulted in a balanced, representative, comprehensive, and high-quality training and testing dataset (Fig. 7A). The dataset was further divided, with 80% allocated for training and 20% reserved for independent testing. The training data was used to train the NetRNApan and DeepM6ASeq models, while the independent testing data was employed to evaluate NetRNApan, DeepM6ASeq [42], and SRAMP [67]. Notably, for CLSM6A [68], which offers cell line and tissue-specific models, we adopted a different validation strategy by extracting positive sites from A549, HeLa, and CD8T cells and matching negative sites as previously described (Fig. 7A).

Using the constructed dataset, we trained and evaluated NetRNApan for predicting m^6^A modifications (Fig. 7A and B). The model was trained as previously described and assessed using 5-fold cross-validation (Fig. 7B). The results demonstrated an area under the curve (AUC) of 0.8710 for the 5-fold cross-validation and a mean AUC of 0.8650 (Fig. 7B), indicating high prediction accuracy. Further validation with an independent dataset, which was not used during training, yielded an AUC of 0.8733 (Fig. 7C). The high AUC values and the consistency between cross-validation and independent validation underscore NetRNApan’s accuracy and robustness (Fig. 7B and C). We compared the performance of NetRNApan with three representative m^6^A site prediction tools: SRAMP, DeepM6ASeq, and CLSM6A [42, 67, 68]. DeepM6ASeq offers two training models: CNN and RNN-CNN. Both models were trained using our dataset and validated with the independent dataset [42]. Among existing tools, DeepM6ASeq CNN-RNN achieved the highest performance with an AUC of 0.8585, followed by CLSM6A CD8T with an AUC of 0.8310 (Fig. 7D). NetRNApan surpassed all other tools and models in performance (Fig. 7C and D). Additionally, we applied NetRNApan to all RRACH sites from human exonic regions and analyzed the topological distribution of predicted m^6^A sites. As depicted in Fig. 7E, the majority of predicted sites were located in the CDS and 3' UTR regions and were notably enriched around the stop codon, consistent with findings from previous studies [69, 70]. In summary, NetRNApan outperforms existing tools in predicting m^6^A modifications, showcasing greater accuracy, robustness, and overall performance (Figs 7B–D)

During the construction of training and testing datasets, we applied a filtering process to exclude all negative instances do not contain the RRACH motif. This ensured that NetRNApan would not learn patterns associated with RRACH, thereby enabling the model to identify sequence patterns around m^6^A sites that extend beyond the RRACH motif. Leveraging this approach, we aimed to investigate the interactions between RBPs and m^6^A-modified sites that do not conform to the RRACH motif. To achieve this, we downloaded RBP binding peaks from 250 eCLIP experiments available from ENCODE [58] and subsequently extracted motifs from these peaks. To align the motif sizes with those derived from the convolutional layers, we fixed the length of the RBP motifs at 10 nt during identification using HOMER [59] (see Methods). By comparing the extracted motifs with those identified by HOMER, we revealed potential regulatory roles of RBPs on m^6^A sites independent of the RRACH motif (Figs 7F–I). For instance, motifs derived from YBX3 (HepG2) binding peaks exhibited a high degree of similarity with motif_201, which was extracted from the convolutional layers of NetRNApan (Fig. 7F and G). Notably, YBX3 has been previously implicated in m^6^A regulation [1, 40]. Similarly, motifs corresponding to NOLC1 (HepG2) and AQR (HepG2) also showed significant similarity with motif_201 (Fig. 7F and G). Furthermore, we observed that m^6^A sites share sequence patterns with several other RBPs, including NIP7 (HepG2), RPS11 (K562), and XRN2 (K562) (Fig. 7H and I). Among these, XRN2 has been reported to require m^6^A modification on nascent RNA for proper transcription termination [71]. While direct interaction between m6A and NIP7 or RPS11 still remains to be explored, both genes were known to be critical for ribosome synthesis and assembly of 40S small ribosomal subunit [72, 73], which was proven to be m6A methylated and can be functional impacted by the modification [74, 75].

In summary, our findings demonstrate that NetRNApan accurately and robustly predicts RNA m^6^A modifications. Additionally, the analysis of convolution layer parameters within the model offers valuable insights into the regulation of m^6^A in humans.

## Discussion

Transcriptome-wide detection of RNA modifications has achieved significant progress and gained substantial interest in the field of genetics and molecular biology. Here, we proposed a novel deep-learning method with interpretable architecture, called NetRNApan, for RNA modification site prediction, motif discovery and trans-regulatory factor identification. Taking experimentally identified m^5^U profiles as a case, we showed that NetRNApan could achieve high accuracy and robust performance with AUC and AUPR values over 0.97. Instead of relying on complex feature coding, NetRNApan extracts important characteristics and crucial genomic sites from primary RNA sequences automatically. In comparison, NetRNApan outperformed 84 conventional machine-learning predictors. More importantly, our method provides both local and global perspectives of the sequence properties, which can aid in understanding the deep learning model. By interpreting the sequence features deciphered by NetRNApan, we identified 135 informative motifs and five representative motif clusters i.e. potential significant for m^5^U modification. The previous computational approaches have limitations and are mostly focused on particular site prediction. By comparison, NetRNApan can identify novel post-transcriptional regulators and 21 RBPs were found, and their binding sites were strongly connected to the extracted top-scoring motifs, such as ANKHD1, RBM4 and HNRNPK. In addition, we employed NetRNApan to systematically scan pathogenic and likely pathogenic single nucleotide polymorphisms (SNPs) associated with cancer, as annotated in ClinVar [76], in order to assess their potential impact on m^5^U modification. By comparing predicted m^5^U profiles of RNA transcripts before and after SNP alterations, we identified three categories of cancer-associated SNPs affecting m^5^U: (i) SNPs that altered the probability of m^5^U modification at existing U sites, (ii) SNPs that generated novel m^5^U sites, and (iii) SNPs that disrupted pre-existing U sites which is probably m^5^ modified. Overall, we identified 43 pathogenic or likely pathogenic SNPs that modified the probability of m^5^U occurrence, 44 SNPs that introduced new m^5^U sites, and three SNPs that abolished existing m^5^U sites (Supplementary Tables S5–S7). Furthermore, we assessed the generalizability of our models by applying NetRNApan to m^6^A prediction tasks. Our model outperformed existing tools in prediction accuracy, as demonstrated by evaluations on independent testing datasets. Additionally, our analysis of sequence patterns surrounding m^6^A sites uncovered potential regulators of m^6^A that operate independently of the RRACH motif.

This study has several limitations. First, the current framework relies exclusively on sequence information. In future work, we aim to integrate additional data modalities, including gene expression profiles and RNA structural features, either to enhance model training or to provide expanded prediction targets. Second, tissue- and cell line–specific predictions were not examined here and will be explored in subsequent studies. Third, our analysis focused on evaluating NetRNApan in the context of m^5^U and m^6^A modifications. Given the increasing recognition of diverse RNA modifications, extending the framework to additional types, such as m^1^A, would further broaden its utility. Moreover, the case study presented to illustrate interpretability, generalizability, and potential for functional investigation was limited to m^5^U profiles across different cell lines. Additional evaluations on other RNA modification types will therefore be important for validating the broader applicability of our tool. Taken together, incorporating multi-omics data, expanding coverage to diverse RNA modifications, and extending analyses across multiple species will represent important directions for the continued development of NetRNApan.

**Key Points**

- We developed NetRNApan, a new deep learning framework for RNA modification site prediction, motif discovery and trans-regulatory factor identification, which overcomes the limitations of existing methods in interpretability and generalizability as well as potential capacity for discovering novel post-transcriptional regulations.
- NetRNApan outperforms 84 traditional machine-learning predictors with more efficient and interpretive feature representations, and identifies consensus motifs potential important for RNA modification.
- NetRNApan revealed promising trans-regulatory factors and provided a protein-binding perspective for understanding the functions of RNA modifications.
- NetRNApan outperforms existing tools in prediction of m^6^A modification and reveals potential RBPs regulates m^6^A independently of the RRACH motif.

## Supplementary Material

Supplementary_material_bbaf690

Supplementary_Table_1_bbaf690

Supplementary_Table_2_bbaf690

Supplementary_Table_3_bbaf690

Supplementary_Table_4_bbaf690

Supplementary_Table_5_bbaf690

Supplementary_Table_6_bbaf690

Supplementary_Table_7_bbaf690

## Data Availability

The data and source code are freely available at GitHub (https://github.com/bsml320/NetRNApan).
