# Supplementary material for: Deciphering RNA modification and post-transcriptional regulation with NetRNApan
Source: Brief Bioinform. 2025 Dec 26;26(6):bbaf690. doi: 10.1093/bib/bbaf690 (PMC12741562; doi:10.1093/bib/bbaf690)
Supplement: Supplementary_material_bbaf690 [file supplementary_material_bbaf690.docx]

**Supplementary data for “Deciphering RNA modification and post-transcriptional regulation with NetRNApan”**

**Supplementary methods**

***Nucleic Acid Composition (NAC)***

The nucleotide frequencies around a specific location were calculated using the NAC encodin. In this instance, NAC encoding determines the frequency for different nucleotide types in a RUN (20, 20). The frequency of the four natural nucleotides ("A", "C", "G", and "U") could be computed as:

$$f\left( i \right)=\frac{n}{N}, i \in\left\{ A, C, G,U \right\}$$

where *N* stands for the length of a RUN (20, 20) and *n* indicates the number of specific types of nucleotides.

***Di-nucleotide Composition (DNC)***

The continuous di-nucleotide pairs in the RUN (20, 20) are calculated using the DNC feature encoding. In DNC feature encoding, there are a total of 16 variables that are described as:

$$Di\left( i,j \right)=\frac{N_{(ij)}}{N-1}, i,j \in\left\{ A, C, G,U \right\}$$

where *N_(ij)_* stands for the number of di-nucleotides represented by nucleotide types *i* and *j*.

***Enhanced Nucleic Acid Composition (ENAC)***

The ENAC encoding determines the nucleotide frequencies using a fixed length (in this example, 5), which moves consistently along with the 5' to 3' terminal direction for every RUN (20, 20). The dimension of the ENAC encoding is defined by the sequence length and the sliding window size, which can be computed as (sequence length - window size + 1 - 4). One definition of the ENAC encoding is:

$$E= \left( b_{1},b_{2}, \ldots, b_{n} \right),$$

$$b\left( i \right)=\frac{N_{(i)}}{N}, i \in\left\{ A, C, G,U \right\}$$

where *N* denotes window size and *n* is calculated by sequence length - window size + 1.

**Supplementary figures**


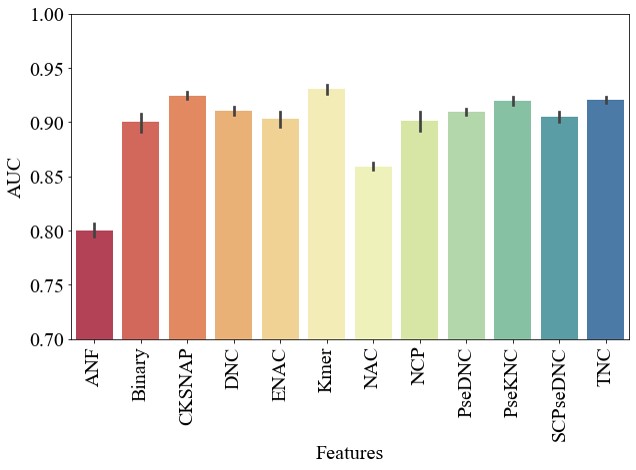


**Figure S1.** Performance of 12 features in RNA modification predictions.


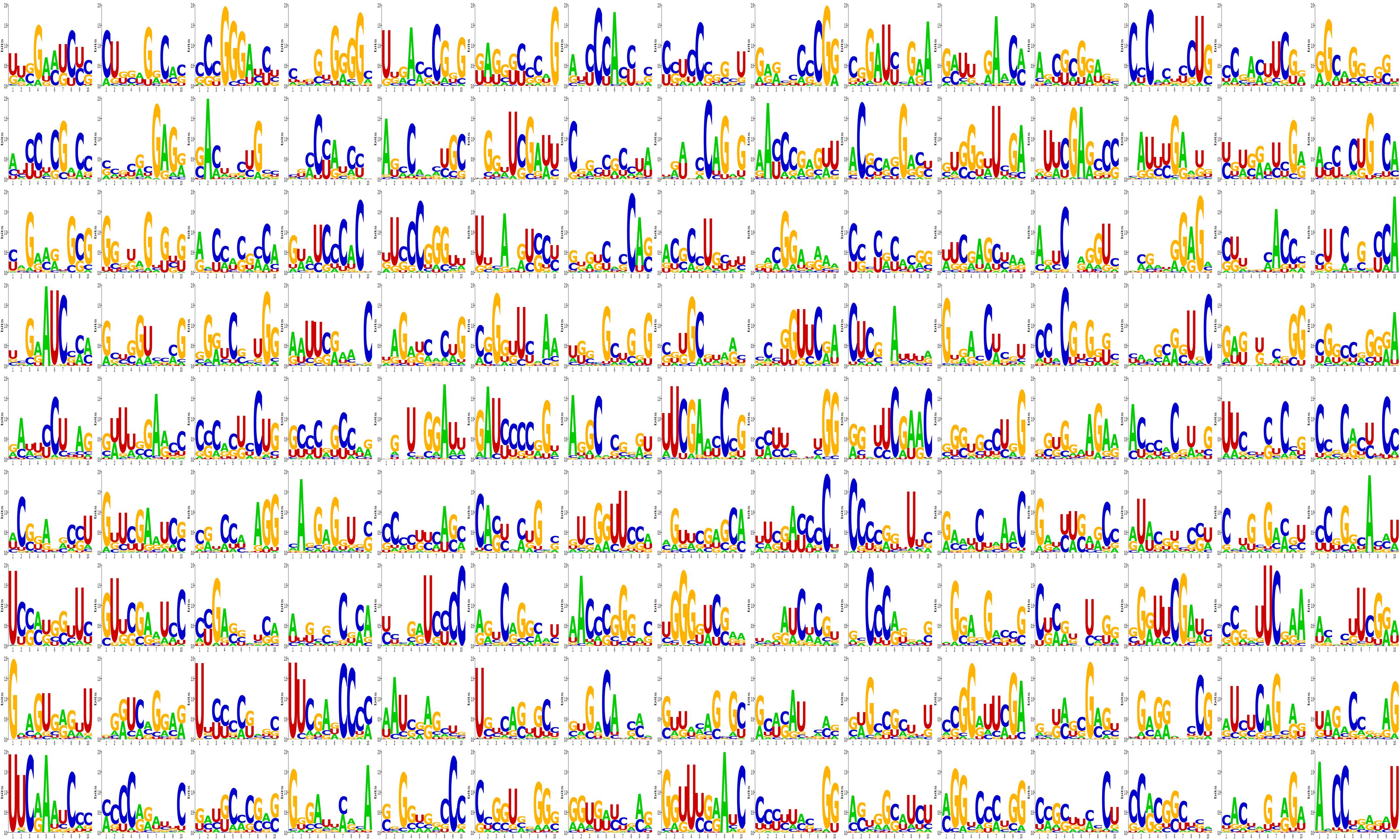


**Figure S2.** Logos of the 50 top-scoring motifs learned from NetRNApan.


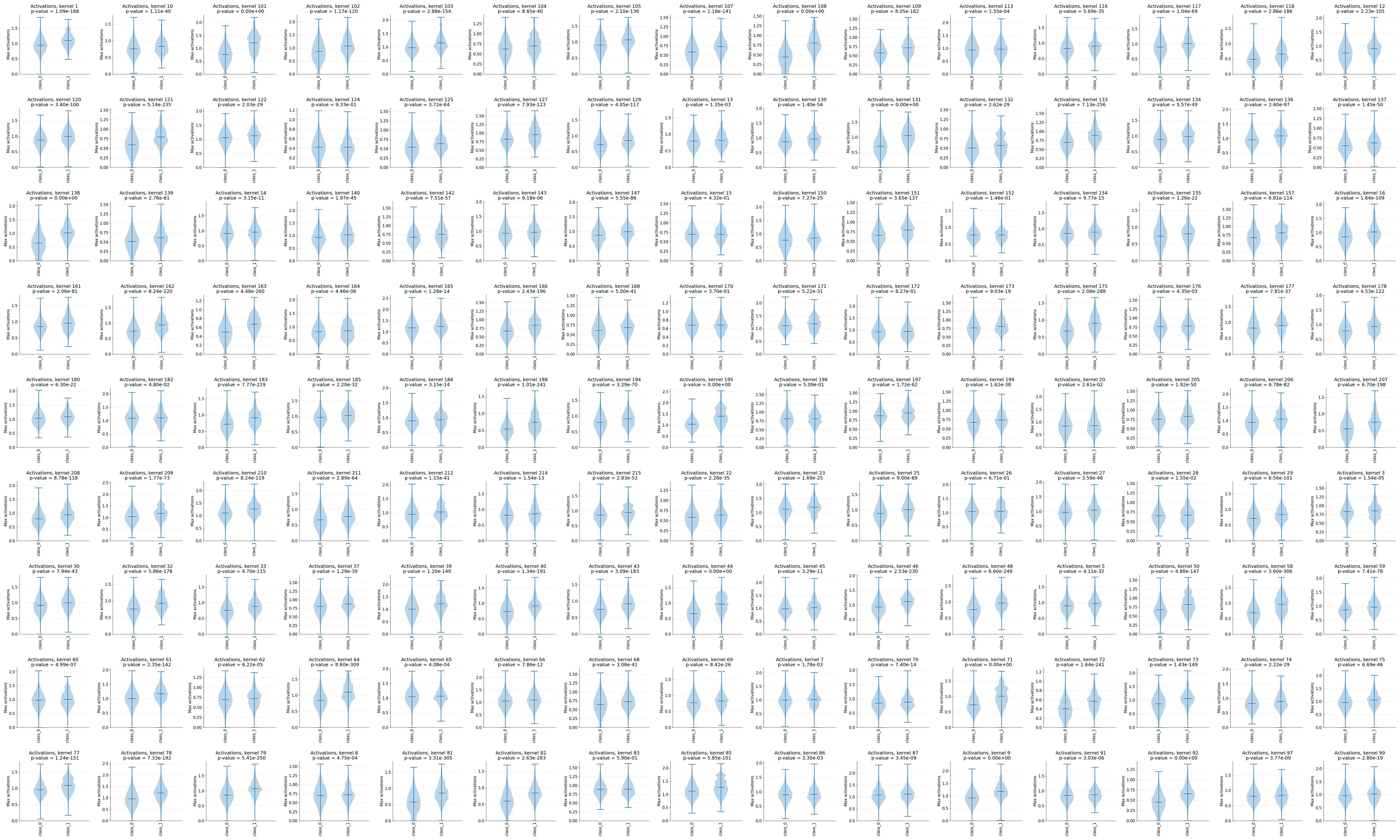


**Figure S3**. Violin plot showed the distribution of the maximum activation scores for motif among modified and unmodified nucleotides.


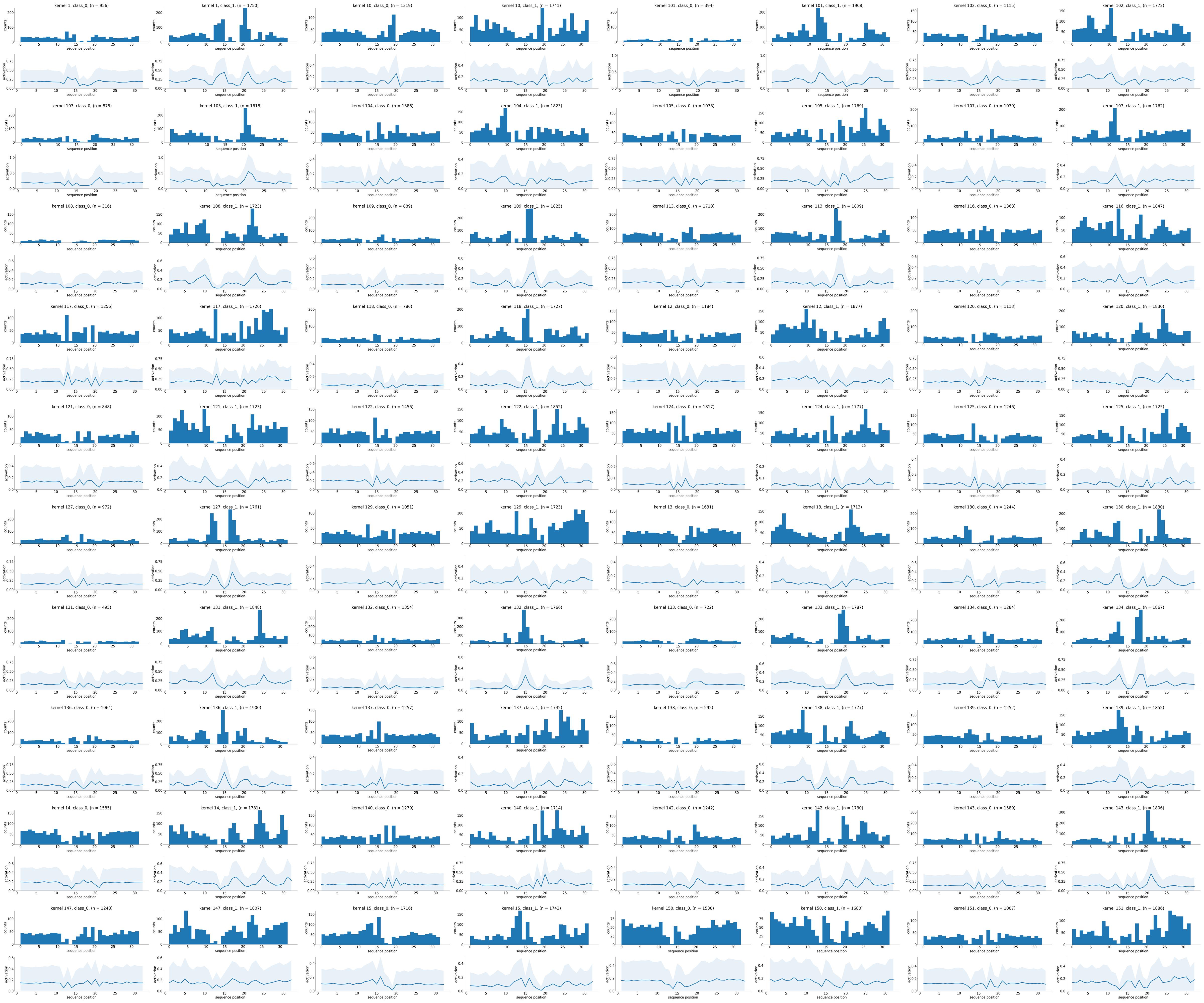


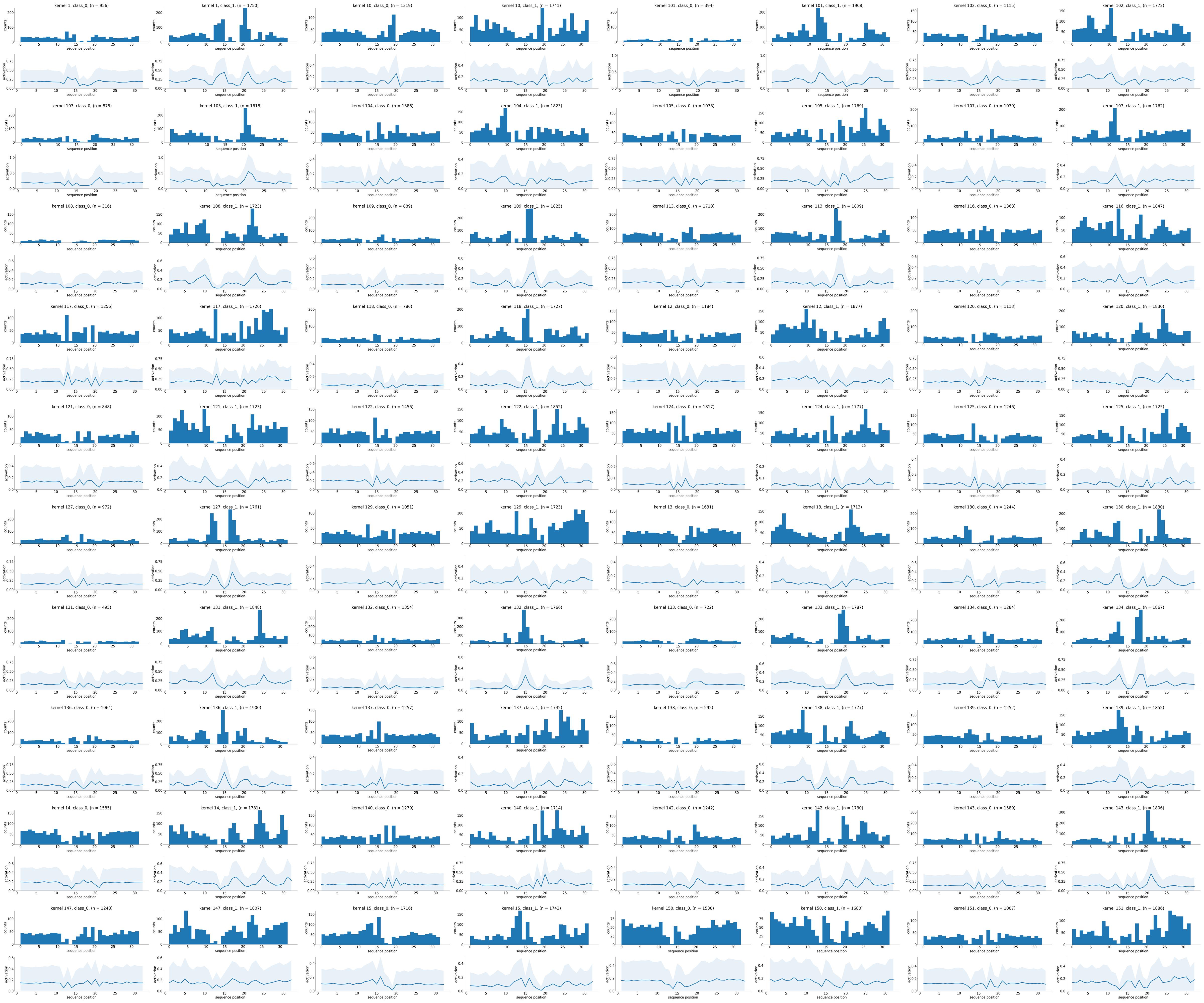


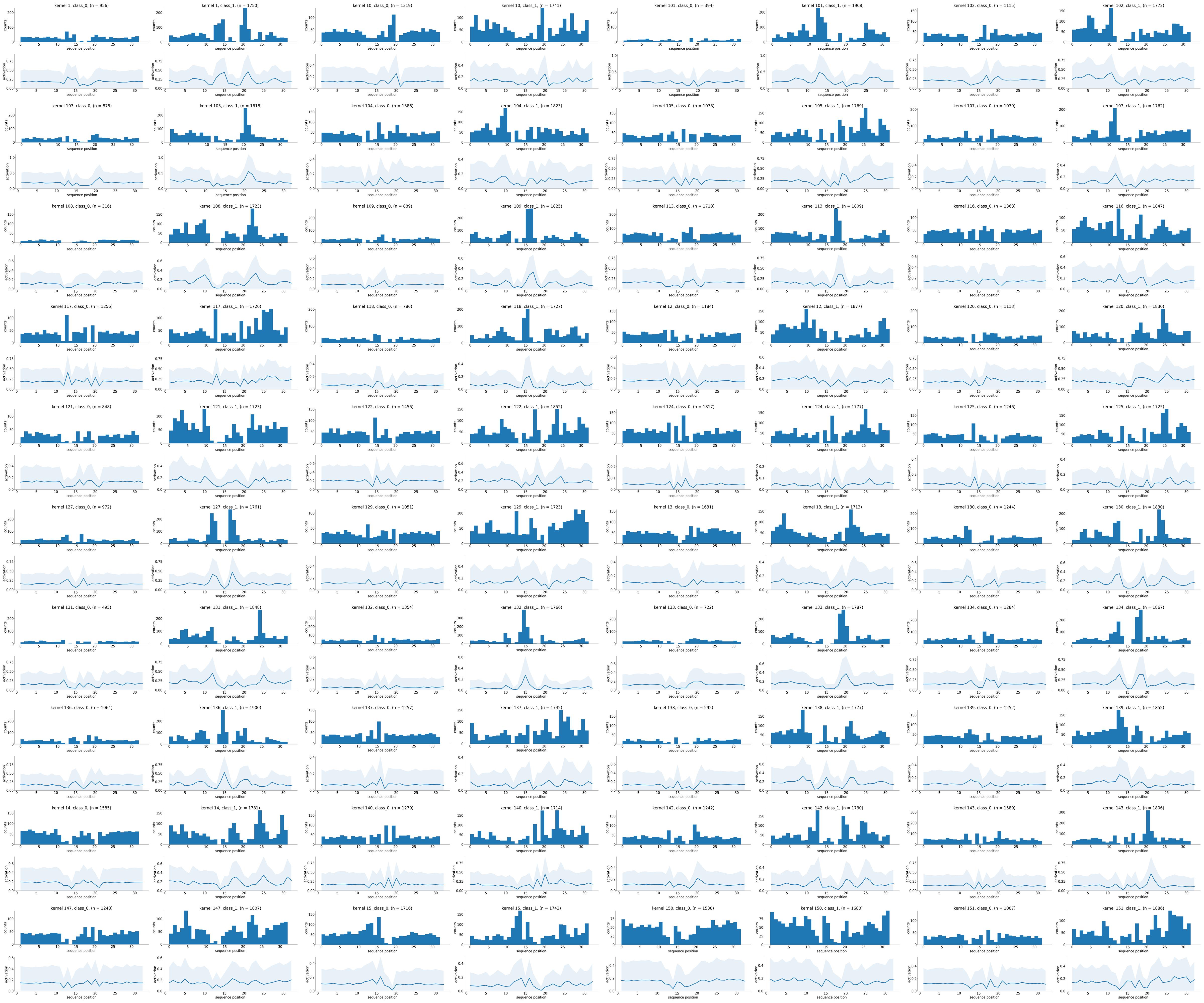


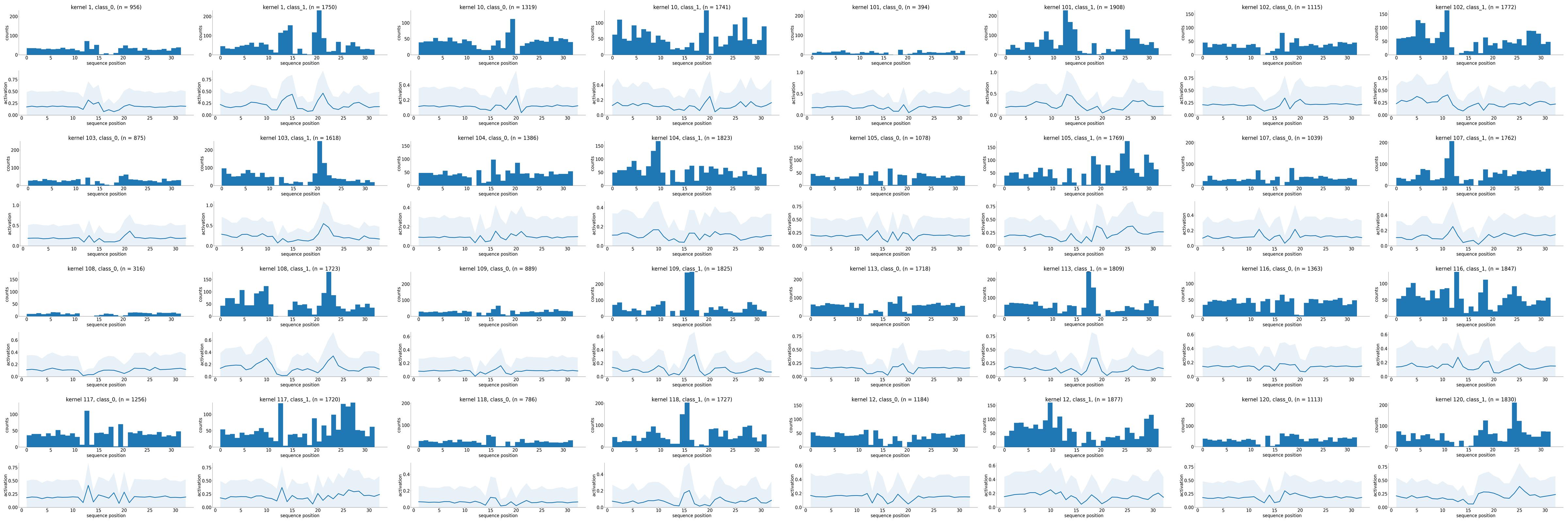


**Figure S4**. Histograms and line charts showed the maximum activation positions where the motif was extracted.

**
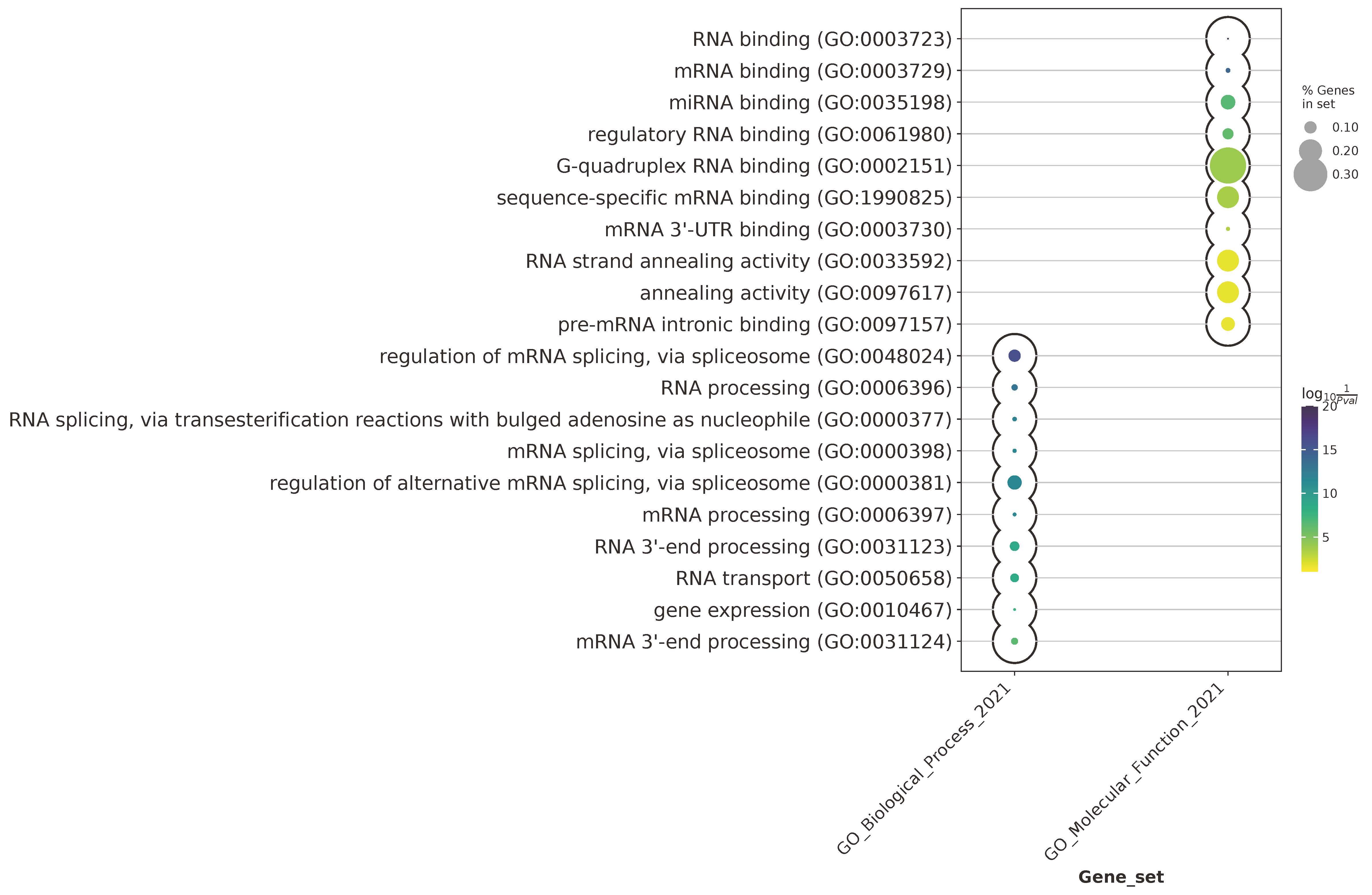
**

**Figure S5.** Functional enrichment analysis of identified RNA-binding proteins (RBPs).
